# Bias of marker genes in PCR of anammox bacteria in natural habitats

**DOI:** 10.1371/journal.pone.0239736

**Published:** 2020-10-01

**Authors:** Min Cai, Fei Ye, Jiapeng Wu, Qihang Wu, Yu Wang, Yiguo Hong

**Affiliations:** Institute of Environmental Research at Greater Bay Area, Key Laboratory for Water Quality and Conservation of the Pearl River Delta, Ministry of Education, Guangzhou University, Guangzhou, China; Chinese Research Academy of Environmental Sciences, CHINA

## Abstract

The identification of anammox bacteria is mostly relied on PCR with various marker genes. However, the community composition revealed by different marker genes and whether the marker genes influence the resulted community composition remain unclear. We compared the community structure of anammox bacteria in enriched and natural environments revealed by 16S rRNA and functional genes (*hzo*, *hzsA* and *hzsB*) from public database and published papers. The genus of *Ca*. *Scalindua* showed the lowest similarities with other genera, especially for the *hzsA* gene (66.9%-68.6%). The 16S rRNA gene is the most commonly used marker gene in natural habitats with 151 out 221 papers in total. The anammox bacterial community composition is distributed according to the source of habitat regardless the use of various marker genes. The role of marker gene is limited with explanatory of 5.4% for variance of community composition, versus 20.5% of habitat. The effect of marker gene is mainly acted on freshwater habitat, which shows significant different community composition revealed by 16S rRNA and *hzo*, with *Ca*. *Brocadia* and *Ca*. *Jettenia* as dominant genus, respectively.

## Introduction

Anammox (anaerobic ammonium oxidation) is the most important finding in nitrogen cycle for the last two decades [1]. Anammox bacteria deeply branch in *Planctomycetales*, and contribute greatly in natural habitats (responsible for 50%, 9~40%, and 4~37% of the nitrogen loss for marine, lakes, and paddy soils) [2–4]. There are five genera of anammox bacteria were identified to date including Ca. *Brocadia* [5], Ca. *Kuenenia* [6], Ca. *Scalindua* [7], Ca. *Anammoxoglobus* [8], and Ca. *Jettenia* [9]. Each genus shows distinct physiological property and adapts to specific habitat [10]. Hence, the community composition of anammox bacteria is very diverse in natural habitats.

The detection of anammox bacteria is greatly relied on PCR based amplification which is an easy, essential, and widely accessible technique [11]. The 16S rRNA gene is the most used marker gene offering phylogeny details in various researches. Besides, many functional genes targeting central anammox activity enzymes were applied for the amplification of anammox bacteria. The functional genes were less used but have high specificity and reflected functional activity [12]. The PCR bias by different maker genes often skews the distribution of PCR products due to the unequal amplification or cloning efficiency [13]. The choosing of marker genes was empiric and varied largely in different habitats in PCR of anammox bacteria. However, how is the community composed revealed by various marker genes, and whether the usage of different marker genes would affect the resulted anammox bacterial community remained unclear. Here we compared the enriched anammox bacteria revealed by different marker genes to show their similarities on genus level. Moreover, the community composition in natural habitats was summarized and compared from published peer-review publications to show the influence of marker genes.

## Methods and materials

The representative sequences of enriched anammox bacteria of each genus was retrieved from GenBank. The similarities of the 16S rRNA and functional genes between genera were obtained by blastx in NCBI and compared pairwisely. Peer-reviewed anammox related papers in natural habitats were searched in Web of Science (published in 2006.10–2020.04) with keywords of *anammox* and *anaerobic ammonium oxidation*. The community composition was summarized according to their original description in each study. The diversity indexes (Shannon Simpson Chao 1 and Ace) were calculated by Mothur program using the collected sequences in each paper (v.1.40.1, https://www.mothur.org/wiki/).

Non-metric multidimensional scaling (NMDS) based on Bray-Curtis dissimilarity was conducted in CANOCO 5 [14]. Similarly, analysis of similarity (ANOSIM) based on Bray-Curtis distances was conducted on community structure difference test using R (version 3.5.1, vegan package). Variation partitioning analysis (VPA) was then used to quantify the relative influences of habitat and marker gene on the anammox bacterial community in CANOCO 5 [14].

## Results

### Comparison of enriched anammox bacteria

**Fig 1:**
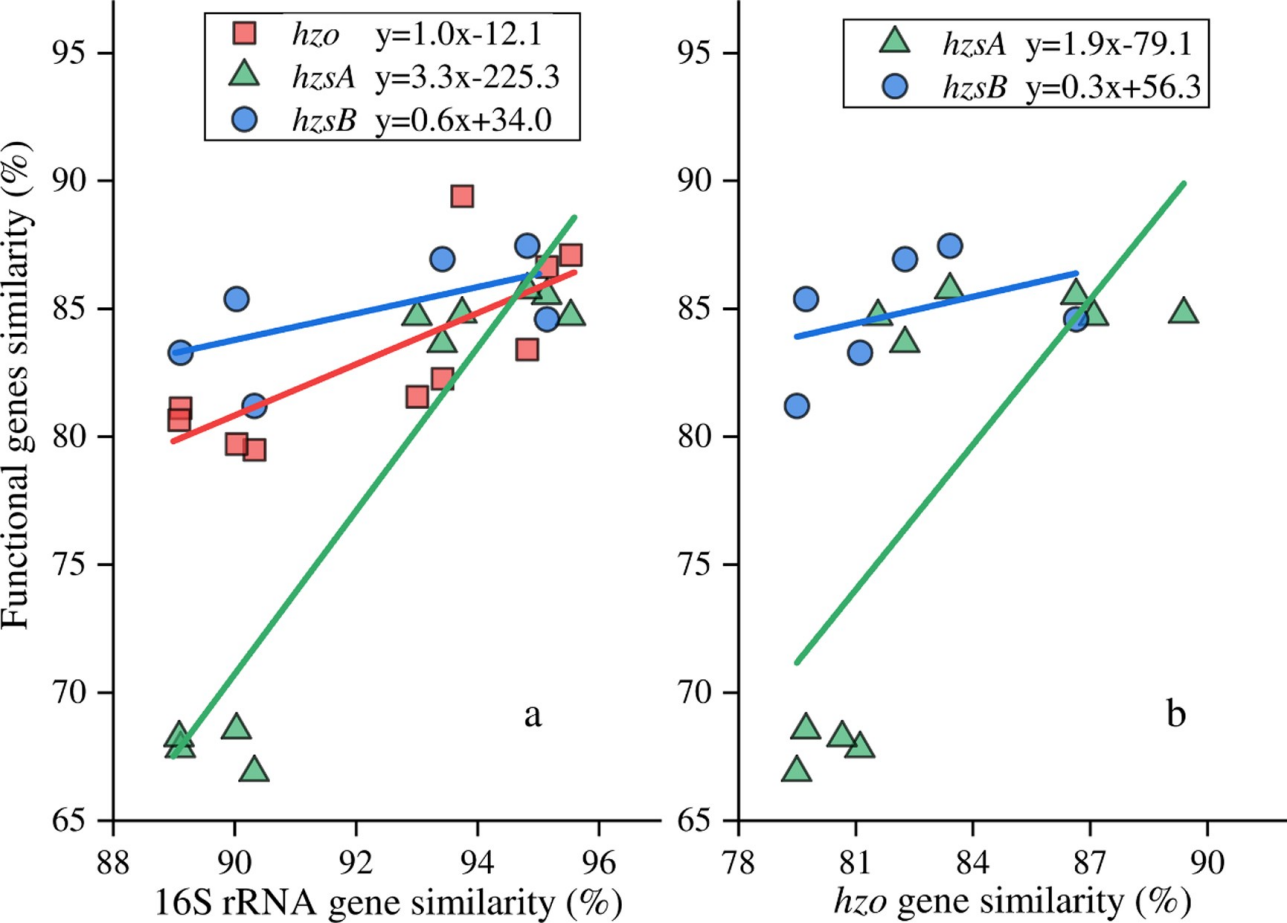
Pairwise nucleotide identity comparisons of functional and 16S rRNA genes of the 5 known genera.

**Fig 2:**
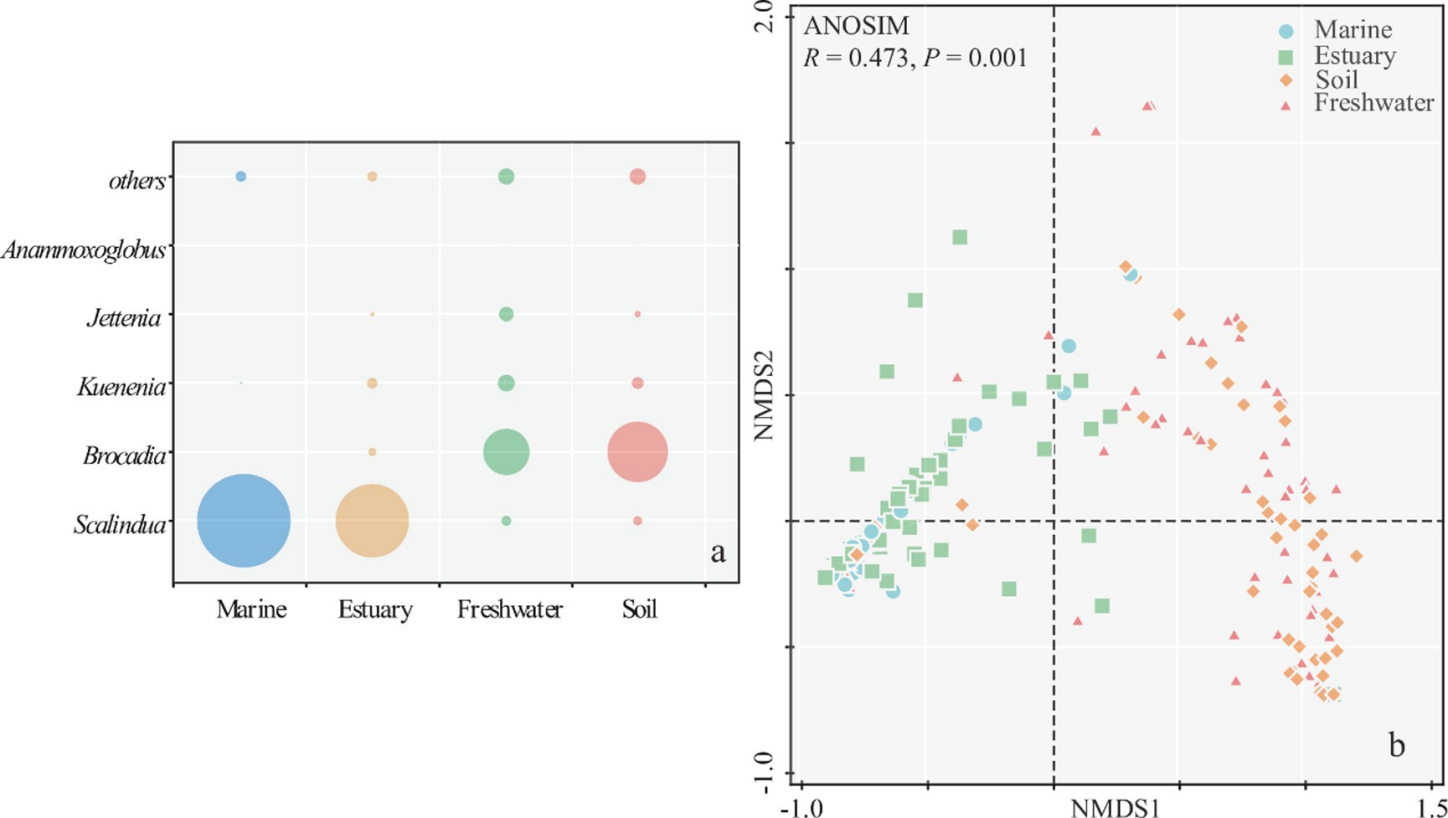
Anammox bacterial community composition in different habitats (a), and NMDS ordination based on Bray-Curtis distance of the sequences affiliation to each genera (b).

Representative nucleotide sequences for the 5 known genera of the frequently used genes: 16S rRNA and functional genes (*hzo*, *hzsA* and *hzsB*) were obtained from Genbank (S1 Table). Sequence of Ca. *Anammoxoglobus* was missing in *hzsB* gene. The nucleotide identities (NI) between the representative sequences of the 5 known genera varied considerably between 16S rRNA and functional genes. The NI of 16S rRNA gene showed the highest values with 90.0% to 95.6% identities, and the *hzsA* gene showed the lowest similarities with NI of 66.9% to 85.7% (Fig 1A). The genus of Ca. *Scalindua* showed the largest difference with other 4 genera in all genes, in which the NI were 89.0%-90.3% for 16S rRNA gene, 79.5%-81.1% for *hzo* gene, 66.9%-68.6% for *hzsA* gene and 81.2%-83.3% for *hzsB* gene. Aside from Ca. *Scalindua*, the other 4 genera showed relatively high similarity with NI of 92.9%-95.6% for 16S rRNA, 81.6%-89.4% for *hzo*, 83.7%-85.7% for *hzsA* and 81.2–87.0% for *hzsB*. The dissimilarities between genera revealed by functional and 16S rRNA genes varied largely except the *hzo* and 16S rRNA genes (Fig 2B).

### Community composition in natural habitats

According to the habitat preference of anammox bacteria, the 221 collected studies were divided into four groups which were marine, estuary, freshwater and soil environments (Table 1). The freshwater environment contained a wide range of habitats including lake, river and freshwater wetland. The 16S rRNA gene was the most used marker gene studying the anammox bacteria which included 151 out 221 studies. The *hzo* gene was the second most used marker gene with 43 studies, and a majority of them were from marine (17) and estuary (14) habitats. Relatively limited studies used the *hzsA* and *hzsB* genes with 10 and 17 studies, respectively.

**Table 1:** Number of collected papers with respective marker genes in each habitat.

	Marine	Estuary	Freshwater	Soil
16S rRNA	34	42	44	31
*hzo*	17	14	8	4
*hzsA*	6	2	1	1
*hzsB*	0	0	4	13

In marine and estuary habitats, the Ca. *Scalindua* was dominant genus accounting for 88.2% and 69.3% of the total anammox bacteria (Fig 2A). In contrast, the Ca. *Scalindua* only accounted for 9.3% and 8.5% in freshwater and soil environments. Meanwhile, the Ca. *Brocadia* dominated these two environments taking for 43.7% and 57.0% of total anammox bacteria, respectively. Sequences of Ca. *Anammoxoglobus* was observed with relatively higher proportion in soil and freshwater environments than those in marine and estuary. Besides, Ca. *Jettenia* and Ca. *Kuenenia* also took higher proportions in freshwater and soil environments with 13.7% and 15.9%, 6.1% and 11.2%, respectively. The NMDS ordination further showed that the anammox bacterial community composition was distributed according to their habitats, in which the *P* value was 0.001 in ANOSIM comparison. The anammox bacteria in marine and estuary environments were closely distributed, and those of freshwater and soil fell together (Fig 2B).

### Community composition revealed by various marker genes

**Fig 3:**
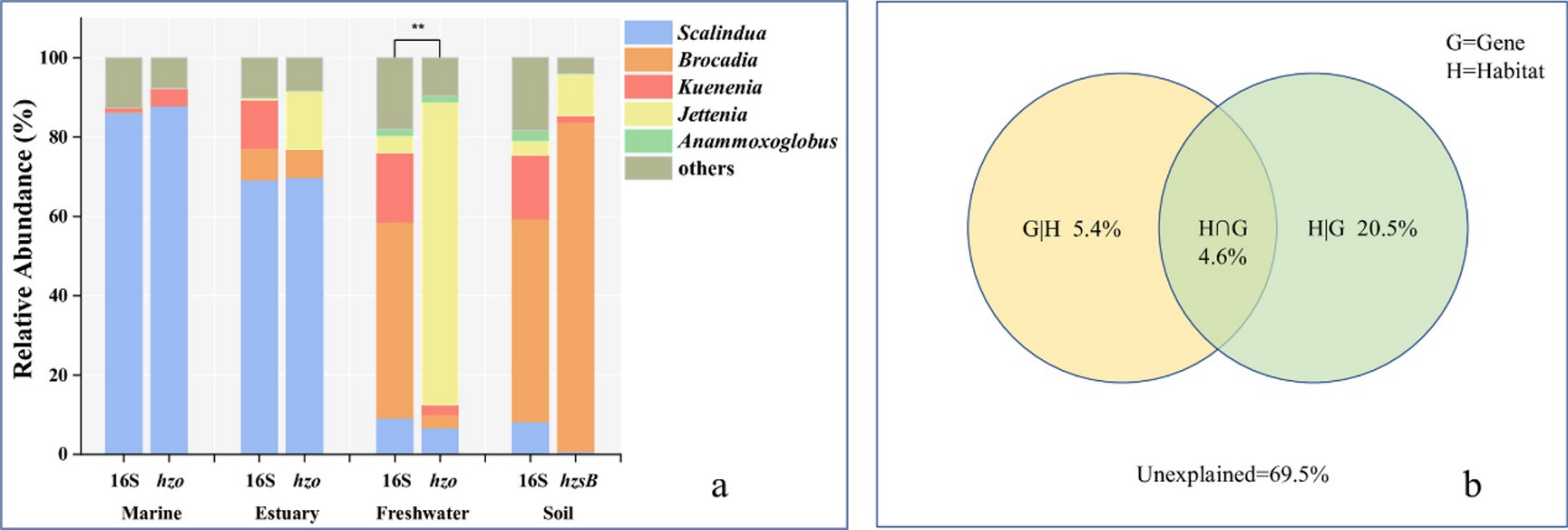
Community composition revealed by the most used two genes in each habitats (a), and Variation Partitioning Analysis (VPA) showing the relative role of habitat and marker gene on the structuring of the anammox bacterial community composition (b).

The community composition revealed by the most frequently used two genes were compared in each habitat to show the effect of marker genes (Fig 3A). The anammox bacterial community in marine environment revealed by 16S rRNA and *hzo* genes were very similar, so were those of estuary environment. The only difference was that the Ca. *Jettenia* revealed by *hzo* gene (14.6%) was more abundant than that of 16S rRNA (0.4%) in estuary. Whereas the 16S rRNA gene identified more Ca. *Kuenenia* related sequences (12.1%) than *hzo* gene (0). However, the community composition in freshwater environment revealed by 16S rRNA differed significantly with that of *hzo* gene. The most abundant genus was Ca. *Brocadia* (49.5%) revealed by 16S rRNA gene, followed by Ca. *Kuenenia* (17.4%) and Ca. *Jettenia* (4.3%). The unclassified sequences name by *others* took a considerable proportion of 18.0%. As to *hzo* gene, the Ca. *Jettenia* was predominant taking 76.3% of the total anammox bacterial community. The other four genus took limited proportion which accounting for 14.2% in total. The unclassified group (9.5%) was smaller than that of 16S rRNA gene. No significant difference was observed in soil habitat using 16S rRNA and *hzsB* genes. In the VPA, the factor of sole habitat took a relatively higher explanation with 20.5% of the variance of community composition. The factor of marker gene explained 5.4% of the variance. The cross effect of habitat and marker genes explained 4.6% of the variance.

### Alpha diversity revealed by different marker genes

**Fig 4:**
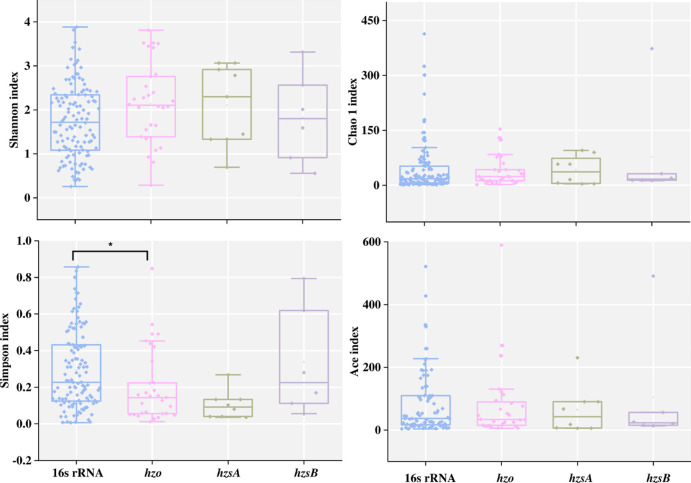
Alpha diversity revealed by the 4 marker genes, significant difference was marked with asterisk (*).

Alpha diversity was assessed by calculating four indexes of Shannon, Simpson, Chao1 and Ace index. The alpha diversity from the 221 studies did not show difference between the 4 habitats (S1 Fig). But the Simpson index revealed by *hzo* gene showed significantly lower values than those of 16S rRNA gene, which suggested higher diversity revealed by *hzo* gene (Fig 4). The other three indexes did not show any difference between the 4 marker genes.

## Discussion

PCR amplification is the primary tool to identify anammox bacteria in natural habitats. Multiple marker genes were applied in PCR along with our increasing knowledge of the evolution and metabolism of anammox bacteria [15]. Previous studies showed that different marker genes might result contrasting community structures [16–18]. However, the bias of marker gene in amplifying anammox bacteria in natural habitats have not been fully investigated.

The genus Ca. *Scalindua* showed the largest difference with other genera presented by all 4 frequently used marker genes, and those amplified by *hzsA* gene showed the highest dissimilarities. In contrast, the dissimilarities between Ca. *Scalindua* and other genera were the lowest presented by 16S rRNA gene. The Ca. *Scalindua* is the dominant genus in marine environment [10]. Therefore, we speculated that the community composition analysis using *hzsA* gene would provide higher resolution when sample from marine was all involved. Besides, the dissimilarities between genera showed comparable values between *hzo* and 16S rRNA genes. It implied a relatively congruent evolutionary relationship of *hzo* and 16S rRNA genes of anammox bacteria.

The 16S rRNA was the most commonly used marker gene for PCR, studies of which were overwhelming (151/221) compared with other marker genes. Besides, most of the cultured anammox bacteria were identified and submitted to public database in form of 16S rRNA gene fragments. There were 17 species of enriched anammox bacteria in GenBank to our statistics [19]. Due to the abundant reference sequences of enriched anammox bacteria, the 16S rRNA gene is a better option in phylogenetic analysis. However, the 16S rRNA gene as a molecular marker is not necessarily related to the physiology of anammox bacteria [12], and the highly conserved nature of the 16S rRNA gene limits its use in revealing the full diversity of anammox bacteria [10]. Analysis of the functional genes involved in specific anammox biochemical reactions could significantly increase the detection efficiency and specificity [20].

The result of anammox bacterial community composition was different using 16S rRNA and *hzo* genes, which revealed more Ca. *Brocadia* and Ca. *Jettenia* in freshwater habitats, respectively. It implied a clear influence of marker genes on the anammox bacterial community composition. The bias of marker genes had been reported that *hzsB* based community analyses showed 3 genera of Ca. *Brocadia*, Ca. *Scalindua* and Ca. *Jettenia* in paddy soil, but the *hzo* gene showed only Ca. *Brocadia* [16]. In marine sediment, the *hzo* gene based diversity analyses revealed 5 genera versus 1 genus by 16S rRNA gene [21]. In a freshwater river, the diversity of anammox bacteria revealed by *hzo* gene was higher than that of 16S rRNA [17]. The contrast of community compositions resulted by different marker genes were mostly caused by the nature 16S rRNA and functional genes which had different detection specificity and efficiency [10]. Besides, the different sources of reference sequences during primers design may lead to bias in amplification efficiency to specific genus [22]. However, no such difference was observed in marine and estuary habitats. It was coincide with previous study that 3 marker genes of 16S rRNA, *hzo*, and *hzsA* all showed that Ca. *Scalindua* was dominant in marine habitats [23]. It was mostly because the diversity on genus level was too low and the genus Ca. *Scalindua* was dominant in these two habitats, that different marker genes resulted similar community composition. It was notable that the marker genes appeared to have no impact on the anammox bacterial community composition in soil, which was coincide with previous study in aquifer sediment using same marker genes [24]. It was probably because that the primers targeting the *hzsB* and 16S rRNA genes have similar specificity and efficiency in amplification. Besides, it also suggested that the role of marker gene in affecting the resulted community composition was limited, as showed in the VPA with low variance from marker gene. Only limited difference was observed on the alpha diversity between marker genes which seemed to be controversial with the community composition analysis. However, we should note that the comparison of alpha diversity was based on sequence level, whereas the comparison of community composition was based on genus level.

In conclusion, we observed the effect of marker gene on the resulted community composition of anammox bacteria in natural habitats, which was significant in freshwater environments. But the overall effect of marker genes was limited for all natural habitats. The 16S rRNA gene showed advance in phylogenetic analysis and the functional genes revealed more diversities of anammox bacteria on genus level.

## Supporting information

S1 Fig Alpha diversity in the 4 types of habitat. (DOCX)

S1 Table Accession number of sequences used in the comparison. (DOCX)
