# Supplementary figures and images for: Bias of marker genes in PCR of anammox bacteria in natural habitats
Source: PLoS One. 2020 Oct 1;15(10):e0239736. doi: 10.1371/journal.pone.0239736 (PMC7529225; doi:10.1371/journal.pone.0239736)

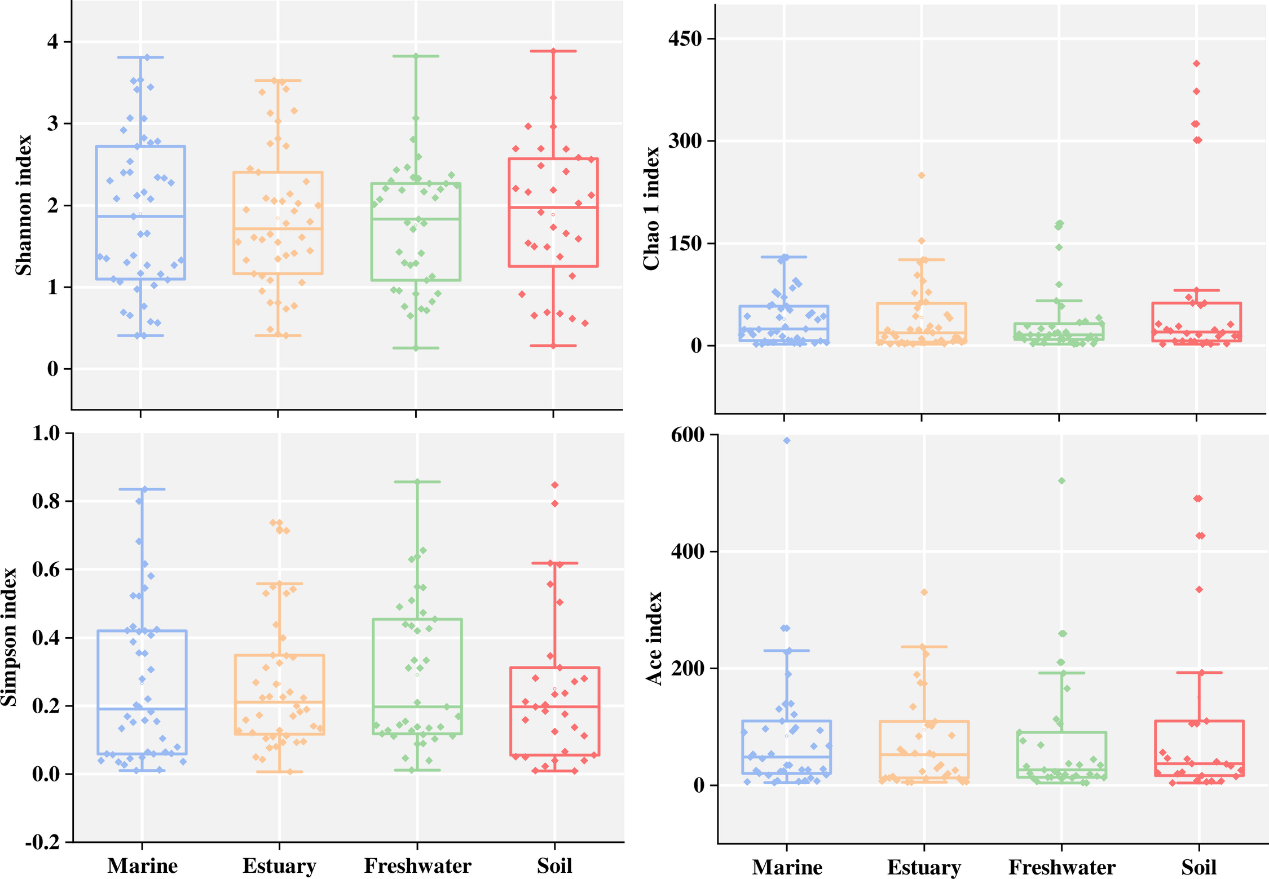


**S1 Fig. Alpha diversity in the 4 types of habitat**

Supplement: S1 Fig — (DOCX) [file pone.0239736.s001.docx]
