# Supplementary material for: Bias of marker genes in PCR of anammox bacteria in natural habitats
Source: PLoS One. 2020 Oct 1;15(10):e0239736. doi: 10.1371/journal.pone.0239736 (PMC7529225; doi:10.1371/journal.pone.0239736)
Supplement: S1 Table — (DOCX) [file pone.0239736.s002.docx]

**S1 Table.** **Accession number of sequences used in the comparison**

|  | *Ca. Scalindua* | *Ca. Kuenenia* | *Ca. Brocadia* | *Ca. Jettenia* | *Ca. Anammoxoglobus* |
| --- | --- | --- | --- | --- | --- |
| 16S rRNA | EU142947 | AF375995 | KY659581 | DQ301513 | DQ317601 |
| *hzo* | FM163627 | FM163630 | FM163628 | EU294365 | FM163629 |
| *hzsA* | JN703716 | CT573071 | JN703714 | JN703715 | JN703712 |
| *hzsB* | JN704476 | JN704475 | JN704473 | JN704474 | - |
